# Genome-Wide Association Study Reveals the *PLAG1* Gene for Knuckle, Biceps and Shank Weight in Simmental Beef Cattle

**DOI:** 10.1371/journal.pone.0168316

**Published:** 2016-12-20

**Authors:** Yuxin Song, Lingyang Xu, Yan Chen, Lupei Zhang, Huijiang Gao, Bo Zhu, Hong Niu, Wengang Zhang, Jiangwei Xia, Xue Gao, Junya Li

**Affiliations:** Laboratory of Molecular Biology and Bovine Breeding, Institute of Animal Science, Chinese Academy of Agricultural Sciences, Beijing, China; China Agricultural University, CHINA

## Abstract

Carcass traits of beef cattle have been genetically improved to increase yield of high quality meat. Genome-wide association study (GWAS) is a powerful method to identify genetic variants associated with carcass traits. For the 770K genotyped SNPs from 1141 Chinese Simmental cattle, we used the compressed mixed linear model (CMLM) to perform a genome-wide association study for knuckle, biceps and shank of beef carcass traits. Seventeen significantly associated SNPs were found, which are located on BTA6, BTA14 and BTA15. Interestingly, one pleiotropic quantitative trait nucleotide (QTN), named *BovineHD1400007259* (*p* < 10^−8^) within the well-known gene region *PLAG1-CHCHD7* on BTA14, was found to govern variation of the knuckle, biceps and shank traits. The QTN accounted for 8.6% of phenotypic variance for biceps. In addition, 16 more SNPs distributed on BTA14 were detected as being associated with the carcass traits.

## Introduction

Genome-wide association studies (GWAS) have enabled us to detect many genetic variants associated with quantitative traits in human [1] and farm animals [2]. The huge number of markers genotyped by the BovineSNP50K Bead Chip [3] provide an ample opportunity for us to perform GWAS in beef cattle. In the past few decades, numerous studies were carried out to map quantitative trait loci (QTLs) responsible for milk production, disease resistance [3, 4], reproduction [5], growth [5–8], meat quality [9–12] and carcass traits [12–15] in cattle. For carcass traits, previous studies had mapped several QTLs for carcass weights on BTA 6, 8 and 14, where one QTN was detected on BTA 6 and related to the encoding of *NCAPG Ile442Met* and the others were identified within gene *PLAG1-CHCHD7* in the region on BTA14 [16]. The association of gene *PLAG1* with carcass weight was found in Hanwoo steers [17]. Gene *NCAPG-LCORL* was found to be associated with carcass traits in multi-breed beef cattle [18]. Both *PLAG1* and *NCAPG* were found to segregate in Japanese Black steers [6]. Meanwhile, *NCAPG* also plays a role in decreasing the subcutaneous fat thickness and increasing carcass yield [6]. Previous studies revealed that five SNPs within or near four genes, including *HS3ST1*, *DVL1*, *PRKCQ* and *HIVEP3*, were significantly associated with eye muscle area, backfat thickness and marbling score [19]. Also, several SNPs in genes *NCAPG* and *LCORL* on BTA6 were found to control hot carcass weight, ribeye area and lower adjusted fat thickness [18]. Several GWAS have been carried out for body compositions in Simmental beef cattle. For instance, the majority of significant SNPs within several haplotype blocks were found to be strongly associated with fore shank weight and triglyceride levels. Moreover, 36 SNPs with high linkage disequilibrium (LD) were detected to be associated with these traits in the *GNAQ* gene [11]. In addition, based on gene set analysis, the gamma aminobutyric acid ergic synapse pathway related to feed intake, weight gain and the non-alcoholic fatty liver disease pathway were found to be responsible for live weight and longissimus muscle area [20].

Statistical power of SNP detection in GWAS can be improved by increasing sample sizes, increasing marker density and choosing advanced statistical methods. In recent years, Bovine 10K, 35K and 77K, Bovine 50K chip had been widely applied for GWAS in cattle [6–10, 13, 16, 18, 19, 21–27]. Since 2014, high throughput Bovine 770K array has been used for GWAS to detect genetic variants associated with intramuscular fat deposition and composition in Nellore cattle [28] and with shank weight and triglyceride levels in Simmental beef cattle [11]. In terms of statistical methods, since PLINK [29] does not consider the confounding effects caused by relatedness among individuals on the power to detect QTL, EMMAX [30] and GAPIT [31] based on mixed linear models have been introduced as standard methods of GWAS for carcass traits [11, 16].

Carcass can be dissected into various body compositions, following the standard of slaughter practice. Body compositions consist of tissue pieces and organs. The separated pieces of meat or tissue not only differ in body part and edible quality, but their qualities are also determined by body morphological structure and construction. The knuckle, biceps and shank are important body compositions of carcass. In this study, we carried out GWAS on separated meat tissue pieces using BovineHD 770k in 1242 Simmental cattle. The objective of this study was to search for QTNs controlling knuckle, shank and biceps in Simmental cattle using the GAPIT software.

## Materials and Methods

### Ethics statement

All procedures strictly followed the guidelines developed by the China Council on Animal Care, and all protocols were approved by the Science Research Department of the Institute of Animal Science, Chinese Academy of Agricultural Sciences (CAAS) (Beijing, China). Samples were collected during regular quarantine inspections on the farms. All farm owners agreed the use of the animals and provided fields for this study.

### Animal resource and phenotypes

The experimental population consisted of 1242 young Simmental cattle born in 2009–2014. This population was derived from the Simmental cattle resource population established in Ulgai, Xilingol league, Inner Mongolia of China (45°N,118°E). After weaning, cattle were moved to Beijing Jinweifuren fattening farm for feedlot finishing under the same feeding and management system. Cattles were well rested for 24 hours before slaughtering to relieve from fear and then made bloodletting after electrical stunning. All measurements of traits were in strict compliance with the Institutional Meat Purchase Specifications for fresh beef guidelines and GB/T 27643–2011. Experimental individuals were slaughtered at 16–18 months of age and at roughly the same time, after which beef carcasses were separated and carcass traits were measured.

For the three carcass traits of interest, knuckle is the quadriceps femoris mucle of beef cattle. Biceps is a piece of bovine fore leg muscle, which is composed of two short-fibred heads separated longitudinally by a thick internal tendon which stretches from the origin on the supraglenoid tubercle to the insertion on the medial radial tuberosity. Shank is meat part along the tibia to the femur in the back of hind leg. During slaughtering, the three traits were dissected following their definitions.

### Sample genotyping and quality control

Collection of blood samples were operated along with regular quarantine inspection on the farms. Genomic DNA was extracted from blood samples using the TIANamp Blood DNA Kit (Tiangen Biotech Company limited, Beijing, China), DNAs with an A260/280 ratio ranging between 1.8 and 2.0 were subjected to further analysis. The IlluminaBovineHDBeadChip with 774,660 SNPs were chosen for individual genotyping. The SNPs were uniformly distributed on the whole bovine genome with a mean inter-marker space of 3.43 kb. SNP chips were scanned and analyzed using the GenomeStudio software package.

For the quality control, individual cattle and their SNP genotypes were handled by the PLINK software package (v1.9) [29]: First, all individuals with missing SNP genotypes ≥ 10% or Mendelian error rate more than 2% were excluded. All SNPs with either less than 90% call rates, minor allele frequencies (MAF) of less than 5%, less than 10% genotype appearances or Hardy-Weinberg equilibrium test p-value < 10^−7^ were removed from the data. Part of the data are uploaded to Dryad, Digital Repository: doi: 10.5061/dryad.4qc06.

### Statistical analysis

#### Phenotypic correction

Prior to GWAS, phenotypes were corrected for some systemic environmental factors using the following linear model,

$$y=\mu +b_{1}\cdot ew+b_{2}\cdot fd+b_{3}\cdot sy+b_{4}\cdot cs+e$$

where *y* is a vector of phenotypic values of the trait under consideration, *μ* is the population mean, *ew* is the live weight of calf entering fattening farm and *b*_1_ is effect of live weight, *fd* is the number of fattening days, *b*_2_ is effect of fattening days, *sy* is slaughtering year, *b*_3_ is the slaughtering year effect, *cs* is calving seasons including the three levels (November to April, May to August and September to October), *b*_4_ is the effect of *cs*, and *e* is a vector of the residuals. The estimated residuals, denoted by y*, were eventually used as the adjusted phenotypic values in the subsequent association study.

#### Association test

To take into account the effects of population stratification and unequal relatedness among individuals, the “Q+K” model of Yu et al. (2011) was used to detect the *i*th QTNs for *i* = 1, …, *N*, where *N* is the number of markers in the genome. The “Q + K” model is

$$y^{*}=Xb_{i}+Qv+Zu+e$$

where y* is the vector of phenotypic values adjusted by non-genetic effects as described early, *b*_*i*_ is additive genetic effect of the *i*th SNP, *X* is a vector of genotype indicator variables whose value is defined as 0, 1, or 2 for the three possible genotypes, G_11_, G_12_ and G_22_, respectively, *v* is vector of population structure effects, *Q* is the principal component matrix calculated from part of the SNPs, *u* is a vector of polygenetic effects assumed to be $N(0,K\sigma_{\alpha }^{2})$ distributed with K being the genomic relationship matrix from all genotyped SNPs and $\sigma_{\alpha }^{2}$ the additive variance, *Z* is a design matrix for the random effects (identity matrix in this case), and *e* is the vector of residual errors following a $N(0,I\sigma_{e}^{2})$ distribution with an unknown error variance $\sigma_{e}^{2}$.

Essentially, the “Q+K” model is a mixed linear model (MLM), where population stratification and SNP effects are treated as fixed effects and the polygenic effects are considered random. GWAS with this model in its original form is computationally very expensive because the mixed model needs to be solved for each SNP tested. By compressing the genomic relationship matrix, MLM has been transformed into a “sire model” that contains only a few effects of individual groups and such a sire model can greatly improve the computational efficiency. The GAPIT software package [31] implemented the “sire model” GWAS using the REML algorithm.

After SNP effects were estimated and their standard deviations calculated by GAPIT, a student-t test statistic was calculated using

$$t_{i}=\frac{\left|{\hat{b}}_{i}\right|}{S_{b_{i}}}$$

for the *i*th SNP, where ${\hat{b}}_{i}$ is the estimated SNP effect and $S_{b_{i}}$ is the standard error. Bonferroni correction for multiple tests was adopted to draw the critical value of the p-value. A SNP was claimed to be significant at the 0.05 genome Type 1 error level if the p-value for the ith SNP was smaller than 0.05/*N*, where *N* is the total number of SNPs. The positive false discovery rate (FDR) across association tests between a SNP and the trait of interest was estimated using the Benjamini-Hochberg method [31]. Finally, the UMD3.1 genome assembly was used to search for genes in the database (http://www.animalgenome.org).

## Results

### Phenotype and quality control

Means (standard deviations), coefficients of variations, heritabilities and pairwise phenotypic correlations between traits (knuckle, biceps and shank) are presented in Table 1. These traits have intermediate heritabilities. These traits are highly correlated with the highest correlation occurring between knuckle and shank. After quality control, seven individuals were removed because they had more than 10% missing genotypes and 94 additional individuals were deleted due to missing phenotypes of the analyzed traits. A total of 59,621, 14,366 and 9,142 SNPs were deleted with call rates < 90%, MAF < 5% and HWE < 10^−7^, respectively. As a result, only 588,150 SNPs on 1141 individuals were used for the GWAS analysis.

**Table 1:** Means (standard deviations), coefficients of variations, heritabilities and pairwise phenotypic correlations between traits knuckle, biceps and shank

Trait	N	Mean (kg)	Coefficient of variation	Heritability	Knuckle	Biceps	**Shank**
Knuckle	1141	9.26(1.47)	6.30	0.44	1		
Biceps	1136	1.07(0.30)	3.57	0.39	0.44	1	
Shank	1141	8.07(1.19)	6.78	0.51	0.87	0.45	1

### Population stratification assessment

**Fig 1:**
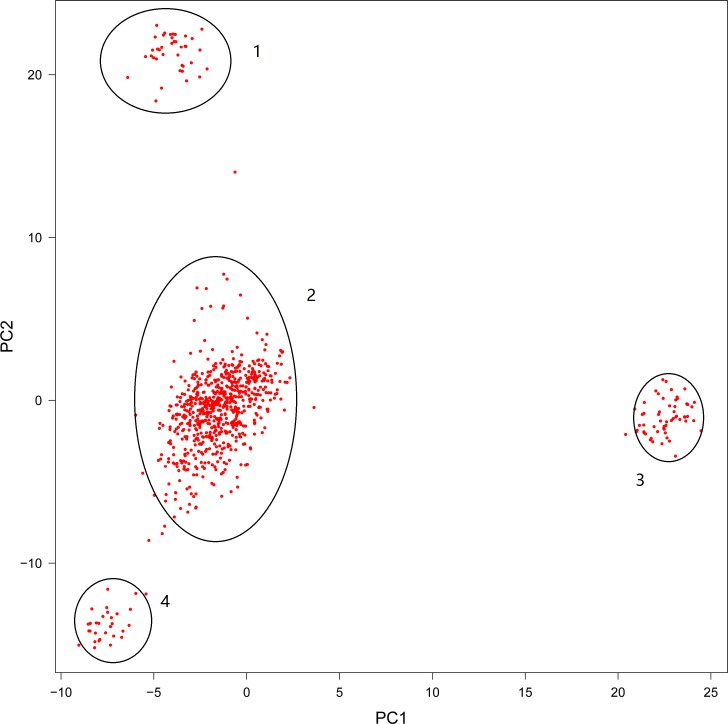
Principal component (PC) plot. The second principal component (PC2) is plotted against the first principal component (PC1). The four separated groups are marked by circles with number 1,2,3 and 4.

Based on the kinship matrix calculated from 50,000 randomly sampled SNPs, the principal component analysis (PCA) showed that the first two principal components (PC1 and PC2) contributed more than 85% of the marker variation. The population was clustered into four groups, as displayed in Fig 1, and hence there was significant population stratification. The first two PCs were considered as covariates and incorporated into the “Q+K” mixed model in our study.

### GWAS analysis

With 588,150 markers, the threshold for the *p*-value was *p* = 0.05/588150 = 8.50×10^−8^ and–log10(*p*) = 7.07 after Bonferroni correction. Fig 1 shows the Manhattan plots of–log10(*p*) against genome locations of the SNP markers. Significant QTNs were claimed at the peaks that passed over the critical value in the Manhattan plot. Based on this criterion, a total of 9 SNPs were significantly associated with the three traits, of which one for knuckle, six for biceps and two for shank. The GWAS results are listed in Table 2. Additionally, 8 SNPs at high peaks whose *p*-values were close to the threshold *p*-value were also considered as candidate QTNs. The FDRs of these QTNs were all < 0.05, except for four QTNs occupying the following four positions: *BovineHD0600009630*, *BTB-00622352*, *BovineHD140000716*1 and *BovineHD1400007161*.

**Table 2:** SNPs identified for Knuckle, biceps and shank.

Trait	Chromosome	QTN	*p*-value	FDR adjust *p*-value	Located gene
Knuckle	14	*BovineHD1400007259*	4.73E-08	0.0317	*PLAG1*
	6	*Hapmap26308-BTC-057761*	2.36E-07	0.0790	*LAP3*
	6	*BovineHD0600009630*	2.36E-07	0.1242	*CCSER1*
	15	*BTB-00622352*	7.52E-07	0.1259	*-*
Biceps	14	*BovineHD1400007259*	2.76E-08	0.0071	*PLAG1*
	6	*BovineHD0600010952*	4.85E-08	0.0071	*-*
	6	*BovineHD0600010950*	5.07E-08	0.0071	*-*
	6	*BovineHD0600010951*	5.40E-08	0.0071	*-*
	6	*BovineHD0600010953*	6.26E-08	0.0071	*-*
	6	*BovineHD0600010956*	7.06E-08	0.0071	-
	6	*BovineHD0600011178*	5.45E-07	0.0367	-
	6	*Hapmap30134-BTC-034283*	1.90E-07	0.0165	-
	6	*BovineHD4100004565*	4.71E-07	0.0357	-
	6	*BTA-75902-no-rs*	7.27E-07	0.0441	*KCNIP4*
Shank	14	*BovineHD1400007259*	9.48E-09	0.0064	*PLAG1*
	14	*BovineHD4100011330*	7.82E-08	0.0262	-
	14	*BovineHD1400007161*	6.88E-07	0.1537	-

#### Knuckle

A total of 4 QTNs were mapped at *Hapmap26308-BTC-057761* and *BovineHD0600009630* on BTA6, at *BovineHD1400007259* on BTA14 and at *BTB-00622352* on BTA15. Only *BovineHD1400007259* was identified as a QTN that is 25 Mb away from the initial marker on BTA14, because the *p*-value exceeded the corrected threshold. The most significant QTN resides within gene *PLAG1*. The two SNPs on BTA6 are candidate QTNs belonging to genes *LAP3* and *CCSER1*, respectively. *BTB-00622352* is located in the region where genes *LOC100848982* and *LOC514367* overlapp. The largest QTN at *BovineHD1400007259* only explained 2.9% phenotypic variation.

#### Biceps

**Fig 2:**
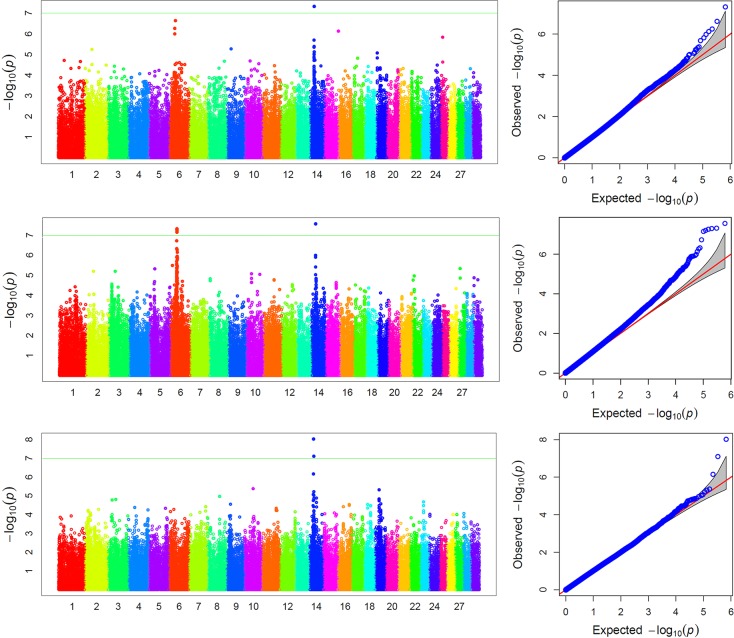
Manhattan plot of -log10 (p) from the beef cattle GWAS for three carcass traits (knuckle, biceps and shank). Chromosomes 1–29 are color coded separately. The horizontal reference line indicated the genome-wise significance levels (–log_10_(*p*) = 7.07).

A total of 10 SNPs were associated with biceps, representing the most SNPs associated with a single trait. Five SNPs on BTA6 and one SNP on BTA14 were identified as the QTNs, while four SNPs as QTN candidates on BTA6. As displayed in the middle panel of Fig 2, five QTNs were mapped at *BovineHD0600010952*, *BovineHD0600010950*, *BovineHD0600010951*, *BovineHD0600010953* and *BovineHD0600010956*. The last one overlaps with a peak on BTA6. Notably, we found *BovineHD1400007259* and *BTA-75902-no-rs* overlapped with genes *PLAG1* and *KCNIP4*. The phenotypic variance explained by the 10 detected SNPs ranged from 0.009 to 0.086.

#### Shank

A QTN mapped at *BovineHD1400007259* resides within gene *PLAG1*. Two other QTNs are 24 Mb and 26 Mb away from the initial marker on BTA 14 and very close to gene *PLAG1*.

In summary, a QTN at *BovineHD1400007259* was found to be simultaneously associated with knuckle, biceps and shank, showing a typical pleiotropic effect of of the QTN in gene *PLAG1*. All detected QTNs contributed relatively low proportions of the phenotypic variation, but QTN BovineHD1400007259 explained over 8.61% of the phenotypic variance for biceps.

## Discussion

In this study, we performed a GWAS for body compositions including knuckle, biceps and shank using high density Bovine HD BeadChip in Chinese Simmental cattle. Previous studies using PLINK [29] have located some QTLs for carcass weight, eye muscle area, back fat thickness and marbling score [19]. Hot carcass weight, adjusted fat thickness, marbling, rib eye area and slice shear force have also been analyzed with PLINK to obtain estimated breeding values (EBV) in genetic evaluation using multivariate animal models [18]. Similarly, polygene effects have also been incorporated into the animal models to control the polygenic background for detection of single SNPs and the interaction between two SNPs to map cold carcass weight, longissimus muscle area, rib thickness, subcutaneous fat thickness, beef marbling standard and carcass yield [6]. The methods adopted above did not consider the effects of the SNPs outside the test SNP on the statistical power of QTL detection. The EMMAX software [30] was therefore adopted to analyze carcass weight [16] by modifying PLINK with genomic best linear unbiased prediction. Notably, causal variants or QTNs associated with knuckle, biceps and shank in Chinese Simmental cattle have been efficiently detected here using the compressed MLM implemented in GAPIT [31].

Importantly, the most significant SNP for knuckle, biceps and shank weights is located within the *PLAG1* locus. Actually, several QTNs have been found to be located in the well-known *PLAG1-CHCHD7* region [5, 6, 13, 16, 17] on BAT14. In human, *PLAG1* is related to the pleiomorphic adenoma gene [32–34] and regulates human height [35]. Additionally, the *PLAG1* knock-out mice are 30% smaller than their wild-type litter mates at birth [36]. Also in cattle, Karim et al.[37] reported two QTNs influencing on bovine stature and mapped them to the *PLAG1-CHCHD7* intergenic region. Carcass weights have been proven to be associated with gene *PLAG1* by GWAS in Japanese Black cattle [16] and Hanwoo steers [17]. In addition to QTNs related to *PLAG1*, some QTNs were found to be responsible for changes in knuckle, biceps and shank in Chinese Simmental cattle, which carry gene *LAP3*, *CCSER1* and *KCNIP4*. As an aminopeptidase that catalyzes the exclusion of amino acids from intracellular proteins and peptides, *LAP3* (*leucine aminopeptidase 3*) is highly articulated in the bovine pineal gland, kidney, skin, intestine, mammary, and adipose tissues (UGID: 1959690, UniGene Bt.56962). *LAP3* has been confirmed to be also associated with milk production and body size in Charolais [37–39]. For the knuckle, biceps and shank traits in Chinese Simmental Cattle, no significant SNPs that harbor the two genes *LCORL* and *NCAPG* have been detected, but two QTN candidates at *Hapmap30134-BTC-034283* and *BovineHD4100004565* for biceps are in high LD with some SNPs within gene *NCAPG*.

## Supporting Information

S1 File GWAS result of knuckle. The GWAS result of the SNPs that are nominally significant (P<0.05) for biceps, including: SNP names, chromosome, position, p-value and FDR.
(CSV)

S2 File GWAS result of biceps. The GWAS result of the SNPs that are nominally significant (P<0.05) for biceps, including: SNP names, chromosome, position, p-value and FDR.
(CSV)

S3 File GWAS result of shank. The GWAS result of the SNPs that are nominally significant (P<0.05) for biceps, including: SNP names, chromosome, position, p-value and FDR.
(CSV)
